# Behavioral and Emotional Problems in Children and Adolescents with Obesity: A Preliminary Report

**DOI:** 10.3390/jcm13020459

**Published:** 2024-01-14

**Authors:** Anna Guerrini Usubini, Michela Bottacchi, Adele Bondesan, Nicoletta Marazzi, Gianluca Castelnuovo, Alessandro Sartorio

**Affiliations:** 1 Psychology Research Laboratory, Istituto Auxologico Italiano, Istituto di Ricovero e Cura a Carattere Scientifico (IRCCS), 20145 Milan, Italy; m.bottacchi@auxologico.it (M.B.); gianluca.castelnuovo@auxologico.it (G.C.); 2 Experimental Laboratory for Auxo-Endocrinological Research, Istituto Auxologico Italiano, Istituto di Ricovero e Cura a Carattere Scientifico (IRCCS), 28824 Piancavallo-Verbania, Italy; a.bondesan@auxologico.it (A.B.); sartorio@auxologico.it (A.S.); 3 Experimental Laboratory for Auxo-Endocrinological Research, Istituto Auxologico Italiano, Istituto di Ricovero e Cura a Carattere Scientifico (IRCCS), 20145 Milan, Italy; n.marazzi@auxologico.it; 4 Department of Psychology, Catholic University of Milan, 20123 Milan, Italy

**Keywords:** cross-informant agreement, adolescents, childhood obesity, child behavior, emotional problems, behavioral problems

## Abstract

Background: Parent–child agreement regarding emotional and behavioral problems in adolescents with obesity was measured. Methods: One hundred Italian adolescents with obesity (36 males, 64 females, mean age ± SD: 15.3 ± 1.61 years, mean body mass index, BMI: 37.9 ± 5.48 kg/m^2^), hospitalized for a 3-week multidisciplinary body weight reduction program at Istituto Auxologico Italiano, Piancavallo-Verbania, Italy, and one of their parents (*n* = 100, 40 fathers, 60 mothers) participated in the study. Achenbach’s Child Behavior Checklist (CBCL) for parents and the Youth Self Report (YSR) for teens were administered. Results: Most of the CBCL and YSR scores were normal, with more borderline and clinical scores being found in CBCL (29% of borderline scores in attention problems, 28% in affective problems, and 26% in ADHD; 32% of clinical scores in affective problems, 23% in withdrawn/depressed, and 22% in anxiety problems and somatic complains subscales) than in YSR (19% of borderline scores in affective behaviors and 17% in somatic complains; 15% of clinical scores in anxiety problems and 17% in withdrawn/depressed subscale). Young females reported greater anxiety problems (*p* = 0.009), oppositional defiant problems (*p* = 0.029), anxiety/depressed (*p* = 0.030), and internalizing problems (*p* = 0.045) than males. Pearson’s coefficients ranged between 0.273 to 0.517. Conclusions: This study provides information on the cross-informant evaluation of psychological profiles with CBCL and YSR in a clinical sample of adolescents with obesity and their parents.

## 1. Introduction

Childhood obesity is a global public health concern, and the prevalence of childhood and adolescent obesity continues to rise. Over 340 million children and adolescents aged 5–19 were overweight or obese in 2016. More recently, the World Health Organization (WHO) has reported that in 2020, 39 million children under the age of 5 and 150 million children aged between 5 and 19 were overweight or obese [1], and most recent estimates pointed out that these numbers are destined to raise further [2].

Pediatric obesity has several adverse consequences on physical and psychological health in childhood and later in life [3]. Children living with obesity may suffer from cardiovascular disease (CVD), asthma, hepatic steatosis, sleep apnea, and type 2 diabetes. Long-term effects of being overweight in childhood are adult obesity, ischemic stroke, joint disease, cancer, coronary heart disease, and other many chronic conditions. From a psychological point of view, children and adolescents with obesity are at risk for many behavioral and emotional problems. Most of them are teased and bullied and may experience social marginalization and discrimination. Such conditions may lead to low self-esteem, and negatively impact their academic and social functioning [4,5]. Several studies also found concerning levels of internalizing problems such as anxiety and depression, as well as externalizing problems including delinquency and rule-breaking behaviors in children and adolescents with obesity [6]. Others have also identified body image difficulties [7].

Childhood obesity imposes individual and social challenges for children and their families. Treatments for the prevention of childhood obesity include both physical activity and dietary habits promotion activities. Parental involvement in lifestyle changes in their children has been identified as an effective technique in the prevention and treatment of obesity [8]. Family-based interventions for obesity in pediatric age specifically address parenting behaviors and strengthen parental involvement in treating children [9]. Clinicians and researchers suggest that intervening in the family system may provide greater change and longer sustainability of change in the child because of the ability of the family to shape the child’s behaviors in daily life [10].

While parental involvement has been identified as a positive strategy to promote healthy behaviors in children and adolescents with overweight and obesity, research has also shown that parents often do not accurately recognize the detrimental health consequences of their children being overweight or obese [11]. Taking into consideration the above statements, the objective of the current study is to explore in-depth the parental perceptions about the psychological conditions of their children with obesity, bringing out the possible similarities or differences in comparison with the individual perception of the adolescents about their own psychological conditions. This report could be significant for informing how professionals can support the whole family in effecting lifestyle changes.

In this study, we aimed to describe the behavioral and emotional problems of adolescents with obesity by comparing parent and child reports on the Child Behavior Checklist for Children (CBCL) and the Youth Self-Report (YSR), respectively, from the Achenbach System of Empirically Based Assessment (ASEBA) for school-age children for assessing emotional and/or behavioral problems.

## 2. Materials and Methods

### 2.1. Participants and Procedures

One hundred Italian adolescents with obesity (36 males, 64 females, mean age ± SD: 15.3 ± 1.61 years, mean body mass index, BMI: 37.9 ± 5.48 kg/m^2^), hospitalized for a 3-week multidisciplinary body weight reduction program at Istituto Auxologico Italiano, Piancavallo-Verbania, Italy, and one of their parents (*n* = 100, 40 fathers, 60 mothers) participated in the study. Participants of the study were recruited at the Division of Auxology, Istituto Auxologico Italiano IRCCS, Piancavallo (VB), a third-level medical and research center for multidisciplinary obesity rehabilitation. Inclusion criteria were (1) being Italian; (2) being aged between 11 and 17; (3) having BMI > 97th centile for gender and chronological age according to the Italian growth charts [12].

Exclusion criteria comprised any form of physical or mental impairment that could compromise participation in the study.

After being informed about the research and after obtaining both written informed assent from the young patients and consent to participate from their parents, participants were screened for the study with a clinical interview. Once the enrollment was completed, selected participants and their parents were asked to provide socio-demographic data and fill in self-report questionnaires.

The current study was approved by the Ethical Committee of Istituto Auxologico Italiano, IRCCS, Milan, Italy (research project code: 01C625; acronym: FATIPSICOB). Research was carried out according to the Declaration of Helsinki and its advancements.

### 2.2. Measures

The Child Behavior Checklist (CBCL) and Youth Self Report (YSR) [13] are measures of child and adolescent emotional/behavioral problems and social competencies that are completed by parents and adolescents, respectively. Both versions are composed of 113 items rated on a three-point Likert scale which assess eight empirically derived syndromes (anxious/depressed, withdrawn/depressed, somatic complaints, social problems, thought problems, attention problems, rule-breaking behavior, aggressive behavior), which are grouped into two general dimensions (internalizing problems and externalizing problems) and six Diagnostic and Statistical Manual of Mental Disorders (DSM)-oriented scales (depressive problems, anxiety problem, somatic problem, attention deficit/hyperactivity problems, oppositional defiant problems, and conduct problems).

### 2.3. Statistical Analysis

A priori sample size calculation was conducted using G*Power 3.1.9.4 for a bivariate correlation normal model. Setting an alpha of 0.05, and a power of 0.85, we found a required sample size of 96.

Descriptive statistics were computed for all demographical, physical, and clinical variables. Categorical variables were presented in frequencies and percentages, while continuous variables were expressed in means and standard deviations. The normal distribution of the variables was assessed with skewness and kurtosis indices. To assess gender differences as well as differences in different rates of obesity (Group 1: BMI SDS 2–2.99 vs. Group 2: BMI SDS > 3) in all the YSR and CBCL variables, independent samples t-tests were run. Cohen’s *d* was used to quantify effect size. Relations between CBCL and YSR subscales were analyzed with Pearson’s correlation. Critical alpha was set to 0.05.

All analyses were performed with Jamovi (The jamovi project, 2021). Jamovi (Version 1.6) [Computer Software] retrieved from https://www.jamovi.org.

## 3. Results

Most of the young participants lived in Northern Italy (86%) in families with low-to-middle socio-economic status (95%) and had a middle school degree (78%).

### 3.1. Youth Self-Report Teens’ Responses

In our sample, most young participants showed normal scores in all the YSR subscales. A total of 19% of the sample reported having borderline scores of affective behaviors, and 17% obtained borderline scores in somatic complains. Clinical scores of anxiety problems were obtained in 15% of the sample, and 17% of participants obtained clinical scores in withdrawn/depressed subscale. Frequencies of normal, borderline, and clinical scores of YSR are reported in Table 1.

We also compared scores in YSR between sexes, different stages of age (group 1: age < 15 vs. group 2: age ≥ 15), and different degrees of obesity (group 1: BMI SDS 2–2.99 vs. group 2: BMI SDS > 3). Results showed that females reported greater levels of anxiety problems (*p* = 0.009), oppositional defiant problems (*p* = 0.029), YSR anxious/depressed (*p* = 0.030), and YRS internalizing problems (*p* = 0.045) than males. No other statistically significant differences in YSR between different stages of age, as well as between degrees of obesity, were found.

Means and standard deviations of YSR between sexes, ages, and BMI of adolescents are depicted in Table 2.

### 3.2. Child Behavior Checklist Parental Responses Related to the Problems of Their Children

Similar to the teens’ responses, most of the parents’ scores at CBCL related to the problems of their children fell into the normal range. However, a greater number of scores that settled in the borderline and clinical ranges were found. In particular, 29% of parents reported borderline scores in attention problems, 28% obtained borderline scores in affective problems and 26% had borderline scores in ADHD. Clinical scores were obtained by 32% of parents in affective problems, and 23% of parents reported clinical scores in withdrawn/depressed subscale. A total of 22% of parents had clinical scores in anxiety problems and somatic complains. Frequencies of normal, borderline, and clinical scores of CBCL are reported in Table 3.

No statistically significant differences in parental responses of CBCL between sexes, different stages of age (group 1: age < 15 vs. group 2: age ≥ 15), or degrees of obesity (group 1: BMI SDS 2–2.99 vs. group 2: BMI SDS > 3) of their children were found.

Means and standard deviations of CBCL between sexes, ages, and BMI of adolescents are depicted in Table 4.

### 3.3. Correlations

As far as correlations between YSR and CBCL are concerned, all the YSR subscales were positively and significantly associated with CBCL subscales with Pearson’s coefficients ranging between 0.273 to 0.517. All the correlations are shown in Table 5.

## 4. Discussion

Obesity in pediatric age is recognized as a global health problem. In response to the increasing prevalence of childhood obesity, an ever-growing number of health promotion and physical activity programs are being proposed [14]. In these programs, parents are considered the critical role models and primary regulators of their children’s health habits [15]. Whether or not parents encourage their children’s involvement in healthy behaviors may lie within the parent’s perception of their conditions. The current study examined the psychological adjustment of adolescents with obesity and explored parent–child agreement in the evaluation of their emotional and behavioral problems.

With regard to the psychological adjustment of adolescents with obesity, the results of the present study showed that most of the sample of adolescents with obesity had normal scores in YSR subscales, with the lowest percentages of normal scores in the affective problems subscale and the highest percentage of normal scores in rule-breaking behaviors subscale. Borderline scores in YSR subscales ranged from a minimum of 3 (thoughts problems) to a maximum of 19 (affective problems). The lowest clinical scores in YSR were obtained in rule-breaking behaviors (3% of the total sample), while the highest percentage of clinical scores in YSR was found in withdrawn/depression (17% of the total sample). This result is in line with the current literature, which suggests an association between depression and obesity in adolescence [16], even if mechanisms underlying depression-obesity relations remain uncertain.

As shown in Table 3, the parent reports were not dissimilar to those of their children, with most of the subscales of CBCL being in normal ranges (anxious/depressed, withdrawn/depressed, somatic complaints, social problems, thought problems, attention problems, rule-breaking behavior, aggressive behavior, internalizing problems, externalizing problems, depressive problems, anxiety problem, somatic problem, attention deficit/hyperactivity problems, oppositional defiant problems, and conduct problems).

This latter finding differs from the literature examining cross-informant agreement between parents and youth, possibly being related to the different study populations. According to previous studies, discrepancies between parents’ and children’s reports are common. More precisely, a recent meta-analysis conducted by De Los Reyes and colleagues [17] found only a modest cross-informant agreement of 0.28 (95% CI [0.22, 0.33]; *p* < 0.001) between parents and children, where a higher cross-informant agreement was for behavior that was more readily observable (i.e., externalizing vs. internalizing problems). In our study, the percentages of normal scores reported by parents were lower than those reported by children in 8 to 14 subscales. By contrast, borderline scores reported by parents in CBCL were higher than those obtained by children in YSR in 13 to 14 subscales. The clinical scores were more frequent in CBCL than YSR in 11 to 14 subscales. On average, parents rated their children as having more severe emotional and behavioral problems than they recognize in themselves. However, correlations between CBCL and YSR were positive and significant, suggesting that YSR/CBCL subscales tended to move in the same directions and thus higher levels of YSR corresponded to higher levels of CBCL. Correlations across CBCL and YSR were similar to those obtained by Sinclair et al., [18] who found an overall level of agreement for the full sample (r = 0.41).

As far as comparisons between sex, age, and degrees of obesity are concerned, no significant differences in YSR were found, with the only exception of anxiety problems (*p* = 0.009), oppositional defiant problems (*p* = 0.029), anxious/depressive (*p* = 0.030), and YRS internalizing problems (*p* = 0.045) in which females reported higher scores than males. These results are congruent with the study by Burt and Neiderhiser, reporting that females develop more internalizing problems than males [19].

In the parents’ reports (CBCL), no statistically significant differences were observed in relation to sex, age, and degree of obesity of their children, suggesting that such factors did not exert an influence on the parents’ reports. This indicates that the perception of parents was independent of the subjective characteristics of their own children.

Limitations of the study include the use of the single-informant measure, the lack of longitudinal data, and the relatively small sample size. Furthermore, the results of the present study cannot be extrapolated to other populations, being obtained in a specific study group of Italian adolescents seeking an in-hospital multidisciplinary body weight reduction program and their parents.

Future replications with a larger sample, additional informants, such as other family members, or teachers, and the inclusion of longitudinal data could improve the value of our results further. In addition, it could be valuable to include additional information on parents psychological adjustment in order to find significant mediators of the parent–child agreement. Despite limitations, this study adds to the literature information on cross-informant evaluation of psychological profiles with CBCL and YSR in a clinical sample of adolescents with obesity and their parents.

## Figures and Tables

**Table 1:** Frequencies and percentages of normal, borderline, and clinical scores of YSR.

Subscales	Normal (n)	Borderline (n)	Abnormal (n)
YSR affective problems	69	19	12
YSR anxiety problems	80	5	15
YSR somatic problems	67	15	8
YSR ADHD	86	7	7
YSR oppositional defiant problems	81	9	10
YSR conduct problems	84	9	7
YSR anxious/depressed	77	10	13
YSR withdrawn/depressed	76	7	17
YSR somatic complaints	78	17	5
YSR social problems	75	12	13
YSR thought problems	90	3	7
YSR attention problems	90	4	6
YSR rule-breaking behavior	92	5	3
YSR aggressive behavior	82	10	8

Note: YSR: Youth Self-Report. ADHD: Attention Deficit Hyperactivity Disorder.

**Table 2:** Means and standard deviations of YSR between sexes, ages, and BMI of adolescents.

	Males (*n* = 36)		Females (*n* = 64)		Group 1 BMI SDS 2–2.99 (*n* = 45)		Group 2 BMI SDS > 3 (*n* = 55)		Group 1 Age < 15 (*n* = 42)		Group 2 Age ≥ 15 (*n* = 58)	
	Means	SD	Means	SD	Means	SD	Means	SD	Means	SD	Means	SD
YSR affective problems	5.39	2.93	8.03	5.22	7.33	4.68	6.87	4.73	6.50	5.10	7.50	4.36
YSR anxiety problems	2.69 *	1.89	4.61 *	2.73	4.47	2.38	3.47	2.74	3.90	2.75	3.93	2.55
YSR somatic problems	2.25	1.92	3.59	2.54	3.29	2.77	2.96	2.10	2.86	2.37	3.29	2.45
YSR ADHD	5.78	2.64	5.63	3.27	6.29	3.24	5.18	2.81	5.83	3.46	5.57	2.73
YSR oppositional defiant problems	3.64 *	1.93	4.14 *	2.14	4.18	1.99	3.78	2.14	3.81	2.14	4.07	2.03
YSR conduct problems	3.75	3.38	3.42	4.74	3.40	3.77	3.65	4.69	4.14	5.34	3.10	3.30
YSR anxious/depressive	4.94 *	3.80	8.84 *	5.58	8.40	5.15	6.65	5.41	6.98	5.80	7.78	5.00
YSR withdrawn/depressive	3.64	3.01	5.86	3.81	5.09	3.40	5.04	3.93	4.50	3.55	5.47	3.76
YSR somatic complaints	3.58	2.64	5.41	3.54	4.98	3.63	4.56	3.11	4.64	3.32	4.83	3.39
YSR social problems	3.75	2.26	6.09	4.03	5.60	3.40	4.96	3.88	5.00	3.87	5.43	3.53
YSR thought problems	2.97	2.81	4.38	4.21	4.02	4.49	3.75	3.20	3.55	4.11	4.10	3.61
YSR attention problems	6.69	2.71	7.30	3.89	7.53	3.67	6.71	3.35	7.19	3.78	7.00	3.33
YSR rule-breaking behavior	3.83	2.99	3.30	4.31	3.42	3.41	3.55	4.25	3.43	4.81	3.53	3.07
YSR aggressive behavior	7.72	4.41	9.17	6.17	9.33	5.39	8.09	5.79	8.76	6.33	8.57	5.10
YSR other problems	8.67	2.24	8.25	3.23	8.49	2.83	8.33	3.00	8.14	3.17	8.59	2.71
YRS internalizing problems	12.17 *	8.16	20.11 *	11.19	18.47	10.55	16.25	11.10	16.12	11.50	18.07	10.39
YRS externalizing problems	11.56	6.78	12.47	9.85	12.76	8.11	11.64	9.45	12.19	10.62	12.10	7.39
YRS total other problems	22.08	6.78	26.02	12.86	25.64	12.07	23.75	10.44	23.88	12.62	25.12	10.10
YRS total	45.81	16.54	58.59	30.22	56.87	27.46	51.64	26.19	52.19	31.68	55.29	22.75

Note: YSR: Youth Self Report. *: significant mean difference (*p* < 0.05). ADHD: Attention Deficit Hyperactivity Disorder; BMI SDS: Standard Deviation Scores.

**Table 3:** Frequencies of normal, borderline, and clinical scores of CBCL.

Subscales	Normal (n)	Borderline (n)	Abnormal (n)
CBCL affective problems	35	28	32
CBCL anxiety problems	57	21	22
CBCL somatic problems	59	20	21
CBCL ADHD	65	26	9
CBCL oppositional defiant problems	82	14	4
CBCL conduct problems	84	8	8
CBCL anxious/depressed	57	25	18
CBCL withdrawn/depressed	62	15	23
CBCL somatic complaints	57	21	22
CBCL social problems	61	25	14
CBCL thought problems	87	10	3
CBCL attention problems	63	29	8
CBCL rule-breaking behavior	92	7	1
CBCL aggressive behavior	78	15	7

Note: CBCL: Child Behavior Checklist for Children. ADHD: Attention Deficit Hyperactivity Disorder.

**Table 4:** Means and standard deviations of CBCL between sexes, ages, and BMI of adolescents.

	Males (*n* = 36)		Females (*n* = 64)		Group 1 BMI SDS 2–2.99 (*n* = 45)		Group 2 BMI SDS > 3 (*n* = 55)		Group 1 Age < 15 (*n* = 42)		Group 2 Age ≥ 15 (*n* = 58)	
	Means	SD	Means	SD	Means	SD	Means	SD	Means	SD	Means	SD
CBCL affective problems	6.33	3.16	7.28	4.23	7.33	4.24	6.62	3.58	6.50	5.10	7.50	4.36
CBCL anxiety problems	3.50	2.37	4.13	2.24	4.31	2.50	3.56	2.07	3.90	2.75	3.93	2.55
CBCL somatic problems	2.39	1.52	3.14	2.25	2.98	2.28	2.78	1.83	2.86	2.37	3.29	2.45
CBCL ADHD	5.75	3.46	5.09	2.82	5.60	3.53	5.11	2.64	5.83	3.46	5.57	2.73
CBCL oppositional defiant problems	3.53	2.12	3.72	2.10	3.69	2.25	3.62	1.98	3.81	2.14	4.07	2.03
CBCL conduct problems	3.89	3.45	2.70	2.77	3.60	3.53	2.75	2.61	4.14	5.34	3.10	3.30
CBCL anxious/depressed	5.33	4.11	7.56	4.65	7.67	4.91	6.02	4.17	6.98	5.80	7.78	5.00
CBCL withdrawn/depressed	4.39	2.89	5.23	3.57	4.71	3.24	5.11	3.46	4.50	3.55	5.47	3.76
CBCL somatic complaints	3.75	2.25	4.73	2.76	4.58	2.77	4.22	2.50	4.64	3.32	4.83	3.39
CBCL social problems	4.03	3.39	5.25	3.50	5.09	3.34	4.58	3.63	5.00	3.87	5.43	3.53
CBCL thought problems	1.78	2.42	2.14	2.08	2.71	2.47	1.44	1.78	3.55	4.11	4.10	3.61
CBCL attention problems	7.11	3.85	6.58	3.48	7.11	3.93	6.49	3.33	7.19	3.78	7.00	3.33
CBCL rule-breaking behavior	3.81	2.81	2.70	2.52	3.42	2.93	2.84	2.42	3.43	4.81	3.53	3.07
CBCL aggressive behavior	7.44	5.89	7.95	4.52	8.27	5.57	7.36	4.56	8.76	6.33	8.57	5.10
CBCL other problems	8.50	2.08	7.75	2.84	7.98	2.66	8.05	2.58	8.14	3.17	8.59	2.71
CBCL internalizing problems	13.47	8.04	17.53	9.12	16.96	9.49	15.35	8.45	16.12	11.50	18.07	10.39
CBCL externalizing problems	11.25	8.16	10.66	6.37	11.69	7.84	10.20	6.29	12.19	10.62	12.10	7.39
CBCL total other problems	21.42	9.00	21.72	9.43	22.89	9.87	20.56	8.62	23.28	12.62	25.12	10.10
CBCL total	46.14	22.23	49.91	20.99	51.53	22.96	46.11	19.93	52.19	31.68	55.29	22.75

Note: CBCL: Child Behavior Checklist for Children. ADHD: Attention Deficit Hyperactivity Disorder; BMI SDS: Body Mass Index Standard Deviation Score.

**Table 5:** Means (SD), means differences, and correlations between teens’ responses to YSR and parental responses to CBCL.

	Means (SD) CBCL Total Sample	Means (SD) YSR Total Sample	Mean Difference (95% CI)	Pearson’s Coefficients	*p*-Value
CBCL—YSR affective problems	6.94 (3.89)	7.08 (4.69)	−0.1400	0.444	*p* < 0.001
CBCL—YSR anxiety problems	3.90 (2.29)	3.92 (2.62)	−0.0200	0.333	*p* = 0.001
CBCL—YSR somatic problems	2.87 (2.04)	3.11 (2.42)	−0.2400	0.321	*p* < 0.001
CBCL—YSR ADHD	5.33 (3.06)	5.68 (3.04)	−0.3500	0.443	*p* < 0.001
CBCL—YSR oppositional defiant problems	3.65 (2.10)	3.96 (2.07)	−0.3100	0.355	*p* < 0.001
CBCL—YSR conduct problems	3.13 (3.07)	3.54 (4.28)	−0.4100	0.291	*p* = 0.003
CBCL—YSR anxious/depressed	6.76 (4.57)	7.44 (5.34)	−0.6800	0.472	*p* < 0.001
CBCL—YSR withdrawn/depressed	4.93 (3.35)	5.06 (3.68)	−0.1300	0.549	*p* < 0.001
CBCL—YSR somatic complaints	4.38 (2.69)	4.75 (3.35)	−0.3700	0.332	*p =* 0.001
CBCL—YSR social problems	4.81 (3.50)	5.25 (3.67)	−0.4400	0.426	*p* < 0.001
CBCL—YSR thought problems	2.01 (2.20)	3.87 (3.82)	−1.8600	0.273	*p* = 0.006
CBCL—YSR attention problems	6.77 (3.61)	7.08 (3.51)	−0.3100	0.482	*p* < 0.001
CBCL—YSR rule-breaking behavior	3.10 (2.66)	3.49 (3.88)	−0.3900	0.386	*p* < 0.001
CBCL—YSR aggressive behavior	7.77 (5.03)	8.65 (5.62)	−0.8800	0.345	*p* < 0.001
CBCL—YSR other problems	8.02 (2.61)	8.40 (2.91)	−0.3800	0.405	*p* < 0.001
CBCL—YSR internalizing problems	16.07 (8.92)	17.25 (10.86)	−1.1800	0.517	*p* < 0.001
CBCL—YSR externalizing problems	10.87 (7.03)	12.14 (8.84)	−1.2700	0.345	*p* < 0.001
CBCL—YSR total other problems	21.61 (9.23)	24.60 (11.18)	−2.9900	0.439	*p* < 0.001
CBCL—YSR total	48.55 (21.41)	53.99 (26.76)	−5.4400	0.400	*p* < 0.001

Note: CBCL: Child Behavior Checklist for Children; YSR: Youth Self Report; ADHD: Attention Deficit Hyperactivity Disorder; SD: Standard Deviation; CI: Confidence Interval.

## Data Availability

Raw data will be uploaded on www.zenodo.org immediately after the acceptance of the manuscript and they will be available upon a reasonable request to the authors A.G.U. and A.S.
